# Evaluating Environmental and Crop Factors Affecting Drone-Mounted GPR Performance in Agricultural Fields

**DOI:** 10.3390/s26061873

**Published:** 2026-03-16

**Authors:** Milad Vahidi, Sanaz Shafian

**Affiliations:** School of Plant and Environmental Sciences, Virginia Polytechnic Institute and State University, Blacksburg, VA 24061, USA; miladvahidi@vt.edu

**Keywords:** radar, sensing, soil, moisture, attenuation

## Abstract

Drone-mounted ground-penetrating radar (GPR) systems offer new opportunities for integrating subsurface characterization into remote sensing workflows. However, the interaction between flight parameters, surface conditions, and vegetation characteristics remains poorly understood. This study investigates the impact of flight altitude, surface topography, crop presence, and canopy water content on the stability and interpretability of GPR signals collected using a drone. Field experiments were conducted under controlled conditions using agricultural plots with variable canopy cover and soil moisture regimes. Radargrams were processed to evaluate signal amplitude, reflection continuity, and attenuation patterns in relation to terrain slope and vegetation structure derived from co-registered RGB drone imagery. The results reveal that lower flight altitudes and smoother surfaces yield higher signal coherence and greater subsurface penetration, while increased canopy water content and biomass reduce signal strength and clarity. Integrating drone-based GPR observations with surface spectral and thermal data improved discrimination between soil and vegetation-induced signal distortions. The findings highlight the potential of drone–GPR systems as a complementary layer in a multi-sensor remote sensing framework for precision agriculture, environmental monitoring, and 3D soil mapping.

## 1. Introduction

Ground-penetrating radar (GPR) is a non-invasive geophysical technique that transmits electromagnetic waves into the subsurface and records the reflections that occur when these waves encounter boundaries with contrasting dielectric properties. The amplitude and travel time of the reflected signals provide valuable information about subsurface structures, moisture content, and material composition. Because of its ability to produce high-resolution data without disturbing the surface, GPR has become an essential tool across a variety of disciplines, including geotechnical engineering, archaeology, environmental monitoring, and agricultural science [1,2]. It has been widely used for locating buried utilities and cavities [3], mapping soil moisture [4,5], monitoring snow and ice thickness [6,7], characterizing root systems [8], and assessing crop yield potential and soil variability [9]. These applications have demonstrated GPR’s versatility and its growing potential for high-resolution, non-destructive subsurface sensing.

In recent years, the rapid advancement of unmanned aerial vehicle (UAV) technologies and lightweight radar systems has driven a new phase of innovation in the use of GPR. Mounting GPR antennas on drones has significantly extended the range and flexibility of subsurface investigations [10,11]. UAV-mounted GPR systems can survey large and difficult-to-access areas efficiently without the need for ground contact, overcoming many of the logistical limitations of traditional ground-based systems. This capability is particularly attractive in agriculture and environmental monitoring, where field-scale mapping of soil and root-zone properties can support precision management practices. By integrating aerial GPR with other UAV-mounted sensors such as RGB, multispectral, and thermal cameras, researchers can now collect complementary information on both aboveground and belowground conditions in a single flight [12,13]. This combination provides new opportunities for comprehensive assessments of soil–plant–atmosphere interactions that were not previously possible using any single sensing technology.

The adoption of drone-based GPR in agriculture and environmental sciences is growing rapidly because of its ability to characterize soil water content, detect subsurface structures, and monitor dynamic processes such as infiltration and root development [14]. Studies have shown that GPR signals at frequencies near 500 MHz can be used to estimate soil moisture with good spatial precision and to map shallow subsurface layers at decimeter-level resolution [15]. Similarly, UAV-borne GPR has been explored for measuring snow thickness and ice-layer stratigraphy, detecting buried objects, and monitoring soil freezing and thawing cycles [16]. These advancements underscore the potential of aerial GPR as a versatile remote-sensing tool for a wide range of scientific and practical applications.

Despite this potential, the transition from ground-coupled to aerial GPR introduces several challenges that affect signal interpretation and accuracy. The presence of air between the antenna and the ground alters electromagnetic coupling and changes the waveform characteristics significantly. Drone motion, flight altitude, orientation, and structural interference further complicate the signal pattern. Moreover, natural field conditions such as vegetation, topography, and soil roughness influence the propagation path and attenuation of radar waves, leading to uncertainties in the recorded reflections [17,18]. These factors introduce considerable complexity in both data acquisition and post-processing, making it difficult to extract consistent and quantitative information from drone-mounted GPR systems. Understanding the mechanisms that govern signal variability under different conditions is therefore critical to improving the reliability and interpretability of UAV–GPR measurements.

Among the factors influencing drone-based GPR signals, four are particularly important: flight altitude, vegetation biomass, topography, and canopy water content. The flight altitude determines the strength of the air–ground coupling and the extent of signal attenuation; higher altitudes result in weaker coupling and reduced reflection amplitude. Vegetation biomass, including leaves and stems, introduces scattering and absorption, which can distort the signal and obscure subsurface features. Variations in topography cause changes in the antenna-to-surface distance along the flight path, modifying signal travel times and amplitudes even when soil conditions are uniform. Additionally, crop canopy water content, which varies with irrigation, growth stage, and plant health, influences the dielectric properties of the vegetation layer and alters the radar wave’s energy distribution. These factors interact in complex ways, and their combined effects on drone-based GPR signals remain poorly quantified, particularly under real agricultural conditions.

Addressing these challenges requires a comprehensive understanding of how each factor modifies the radar waveform and how their combined effects can be distinguished from true subsurface variations. Despite the increasing number of drone–GPR studies, most previous research has focused on demonstrating feasibility or on developing data processing workflows rather than systematically analyzing the physical factors that alter signal characteristics. Consequently, the interpretation of UAV–GPR data in vegetated agricultural fields remains uncertain, limiting its broader application for soil and crop monitoring.

In this study, we investigate how these four factors—altitude, topography, vegetation biomass, and canopy water content—affect the amplitude and shape of signals recorded by a 500 MHz drone-mounted GPR system. Three separate field experiments were designed to isolate and evaluate the effects of these factors. The first experiment examined how varying the flight altitude alters signal energy and attenuation over a uniform, bare soil surface. The second experiment investigated the influence of vegetation by comparing signals collected over maize plots with different canopy densities during early and mid-growing stages. The third experiment focused on the effect of crop water content by comparing GPR signals collected under irrigated and non-irrigated conditions. These experiments were conducted in realistic agricultural environments to ensure that the findings reflect practical field conditions rather than idealized laboratory setups.

Beyond analyzing raw waveform behavior, this study applies several advanced signal transformation techniques to enhance feature extraction and to highlight how different processing approaches can improve signal interpretation. These include the Laplacian of Gaussian (LoG), Difference of Gaussian (DoG), Morlet-based continuous wavelet transform, and Hilbert–Huang transform. Each of these techniques emphasizes different aspects of the GPR signal, such as edges, frequency localization, and amplitude envelopes, thereby helping to reveal the structure of subsurface reflections and their interactions with the vegetation canopy. Through comparative analysis of these transformations across different experiments, we evaluate their effectiveness for isolating signal components related to specific physical factors. This research contributes to a better understanding of how environmental and operational parameters influence UAV–GPR signal characteristics in agricultural fields. By identifying the relative importance of altitude, canopy presence, topography, and biomass water content, we provide a foundation for developing improved correction algorithms and calibration strategies for drone-based GPR data. The outcomes of this study are expected to support the advancement of UAV-mounted radar systems toward reliable, quantitative monitoring of soil and crop dynamics, enabling broader applications in precision agriculture and environmental sensing.

## 2. Background Principles of Drone-Based GPR Measurement

### 2.1. Fundamentals of GPR Signal Generation

GPR is a pulsed electromagnetic technique. A transmitter creates a very short voltage pulse that drives a broadband antenna; the resulting wavefront expands spherically through the surrounding medium. Whenever that wave meets a boundary where the dielectric permittivity changes air to foliage, foliage to soil, or one soil layer to another, a portion of the energy is reflected toward the receiver, while the remainder continues downward. The receiver converts the returning field into a voltage, amplifies it, and digitizes the waveform so that each sample represents the field strength at a precise instant after launch. Because the speed of propagation depends on permittivity, the travel time of each reflection encodes depth, and its amplitude encodes the dielectric contrast (which, in soils, is dominated by water content). The free-space component of this process is described by the spreading relation [19]:

(1) $$E_{i}\left(t,R\right)=\frac{V_{Tx}\left(t\right)G_{ant}}{4\pi R^{2}}$$

where $G_{ant}$ is the antenna gain, and *R* is the instantaneous path length. Inside a medium of relative permittivity, $\varepsilon_{r}$, the wave slows to $v=c/\varepsilon_{r}$; the two-way travel time to a reflector at depth *d* is therefore [19,20]:

(2) $$t=\frac{2d\sqrt{\varepsilon_{r}}}{c}$$

Reflection strength is controlled by the Fresnel coefficient [20].

(3) $$\Gamma =\frac{\sqrt{\varepsilon_{r,2}}-\sqrt{\varepsilon_{r,1}}}{\sqrt{\varepsilon_{r,2}}+\sqrt{\varepsilon_{r,1}}}$$

So, increases in water content, whether in leaves or soil, raises $\varepsilon_{r}$ and produces stronger echoes. After travelling downward and back upward, the voltage that reaches the receiver in a bistatic pair can be approximated by [21,22]:

(4) $$V_{Rx}=\frac{V_{Tx}G_{ant}^{2}\lambda \Gamma }{{\left(4\pi \right)}^{\frac{3}{2}}R_{air}(2H)}$$

where λ=c/f is the wavelength of at the center frequency f, $R_{air}$ is the fixed separation between the transmit and receive antennas, and H is the antenna height above the ground. The front end analogue applies a known gain $G_{amp}$, and the amplified waveform is digitized against a reference voltage $V_{ref}$.

**Figure 1:**
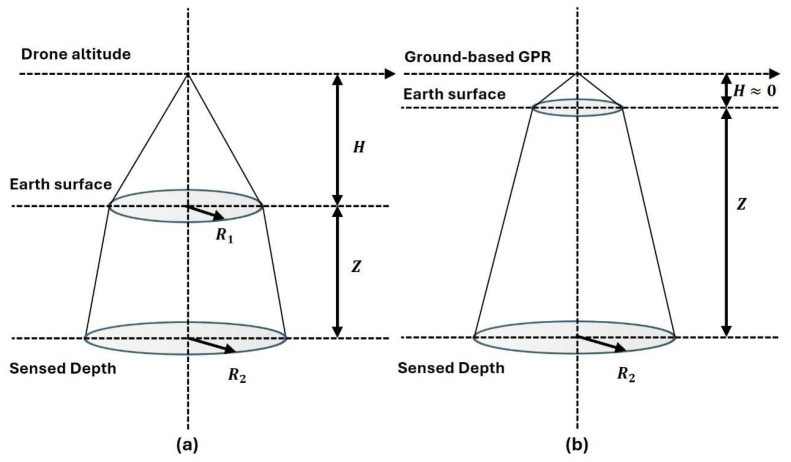
Main-lobe geometry for (**a**) drone-mounted and (**b**) ground-based GPR. $R_{1}$ and $R_{2}$ denote footprint radii at the soil surface and at depth Z; H is antenna height.

Mounting the radar on a multirotor transforms the geometry (Figure 1). The antennas are lifted several meters above ground, converting the survey from a ground-coupled to an air-coupled configuration. Free-space propagation now dominates the first leg of the path, the footprint on the surface widens with altitude, and the returned signal must traverse the vegetation canopy twice. Any objects (vegetation or crop structure) will be an interface, with certain Reflection strength, reflecting and scattering a transmitted pulse. However, given the new geometry of the system, horizontal and vertical resolutions, amplitude intensity, and attenuation rate of signal are affected by drone altitude and can be formulated as follows:

The conical main lobe defines a surface footprint radius:

(5) $$R_{1}=\frac{0.075}{f}+\sqrt{\frac{0.075H}{f}}$$

And a deeper footprint at depth of Z:

(6) $$R_{2}=\frac{0.075}{f}+\sqrt{\frac{0.075}{f}(\sqrt{H}+\sqrt{\frac{z}{\sqrt{\varepsilon_{s}}}})}$$

With f is GHz, the coefficient 0.075 is an empirical constant that comes from the standard beam-footprint approximation for air-coupled GPR antennas, and all lengths in meters. For vertical resolution, the thinnest layer distinguishable in a trace is:

(7) $$D_{Z}=\frac{0.15}{f\sqrt{\varepsilon_{s}}}$$

For the Zond’s 0.5 GHz center frequency at H = 2.5 m, which we flew over the growing season in sandy soil ($\varepsilon_{s}\approx 4$), these relations predict $R_{1}\approx 0.36\;m$, $R_{2}\approx 0.45\;m$ at 0.4 m depth, and $D_{Z}\approx 0.08$ m, dimensions that guided our 1 m × 1 m ground truth frames at 10 cm depth bins. Altitude variation or drone flight altitude tolerations are other factors affecting signal amplitude. For a tolerance of less than 10 cm, the altitude normalization can be neglected; however, for multi-elevation drone flights, the received power falls off as $1/H^{2}$, and the corresponding amplitude scales as [23]:

(8) $$\frac{A}{A_{o}}=K\frac{1}{H}$$

where $A_{o}$ is a reference amplitude at height $H_{o}$, and K lumps antenna and reflection constants [24]. Multiplying each trace by $H/H_{o}$ therefore brings all flights to the same amplitude baseline before feature extraction.

### 2.2. GPR-Mounted Drone Configuration

**Figure 2:**
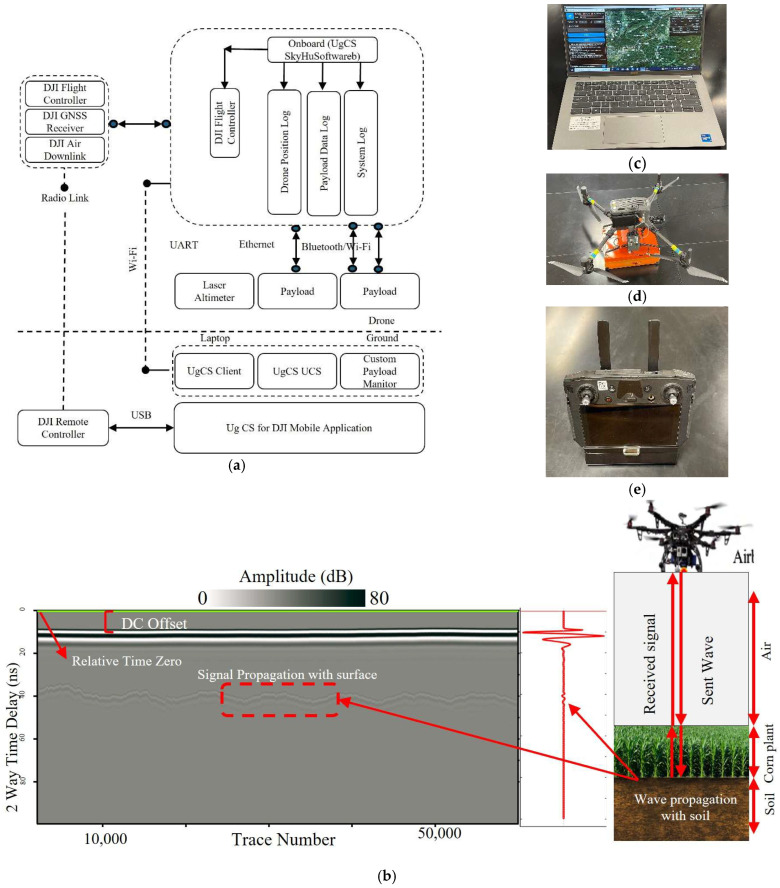
End-to-end drone-mounted GPR system. (**a**) Data-flow diagram linking the DJI M300 flight controller, the onboard UgCS SkyHub computer, the Zond Aero 500 payload and laser altimeter, the DJI remote controller, and the UgCS ground station software 4.9.817. (**b**) Example radargram acquired. (**c**) Ground station laptop running UgCS Client and UgCS UCS for mission planning, telemetry logging, and live quality control. (**d**) DJI M300 airframe carrying the Zond Aero 500 and the UgCS SkyHub payload hub. (**e**) DJI Smart Controller running UgCS Mobile, used to upload missions, monitor flight status, and relay commands between the laptop and the drone.

The sensing system joins three layers: airframe, payload, and ground station, into a single feedback loop, as shown in Figure 2. At the top of the chain is the DJI M300, whose flight controller and multi-constellation GNSS receiver hold the aircraft on the waypoints designed in UgCS. Real-time pose information travels over the factory radio link to the ground and is echoed upward to the onboard UgCS SkyHub computer so that every GPR trace is stamped with centimetre-scale position and attitude. SkyHub, housed beside the Zond Aero 500, acts as a payload hub. It receives trigger commands from the flight controller, pushes them to the GPR over a dedicated Ethernet line, captures the returning traces, appends laser altimeter height supplied on a UART port, and writes three time-synchronised files: a drone-position log, a payload-data log, and a system-health log. The same packets stream down to the ground station through a short-range Wi-Fi bridge, giving the operator a live view of trace throughput and altimeter performance.

The airborne survey relies on a tight handshake between hardware in the air, a handheld controller, and a ground laptop, the three components shown in Figure 2. The DJI M300 in panel (d) lifts the Zond Aero 500 and the UgCS SkyHub computer to altitudes at 2.5 m. SkyHub draws power from the drone, listens to flight-controller triggers, and, in real time, attaches latitude, longitude, height, pitch, roll, and yaw to every trace that streams in over Ethernet from the GPR. A laser altimeter, wired on a UART line, adds a centimetre-level height measurement that later feeds the amplitude-normalization step described in Section 2.1.

During pre-flight, the operator opens UgCS Client on the ground station laptop, panel (c). Waypoints are laid out parallel to the crop rows, and flight speed, height, and trace spacing are set to match the spatial resolution predicted by Equations (6)–(8). The compiled mission is pushed through a USB cable to the DJI Smart Controller, panel (e), which runs UgCS Mobile. When the mission starts, UgCS Mobile relays each waypoint to the M300 over the built-in radio link and at the same time forwards telemetry back to the laptop via Wi-Fi, where UgCS UCS writes a second copy of the position log for redundancy. Custom-payload messages ride the same channel, so the ground station can pause data collection or change trace spacing without recalling the drone. The moment the flight controller reaches a trigger point, it instructs SkyHub to fire the radar. The Zond Aero 500 emits a sub-nanosecond pulse, records the returning field, and sends the digitised trace back to SkyHub. The annotated radargram in panel (b) comes directly from that real-time stream. The top few samples mark time-zero after DC-offset removal; the bright line below is the canopy-plus-surface return, and the gradually fading texture beneath carries the information used later for subsurface monitoring purposes.

### 2.3. Anatomy of a Trace: Direct Air Wave, Canopy/Surface Cluster, Subsurface Window

**Figure 3:**
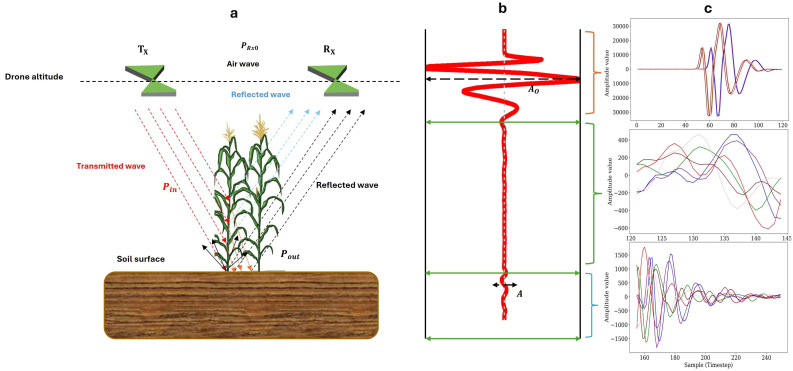
The overal overview of GPR-drone, corn crop and signal propegation with crop, surface and soil subsurface (**a**). The anatomy of a single trace and its position in an A-scan radargram (**b**). The red band is the direct air wave used for time-zero alignment; the green band is the canopy/surface cluster that evolves with biomass and surface moisture; the blue band is the subsurface window (**c**).

Figure 3 presents an annotated radargram acquired by our drone-mounted GPR, plotted in a vertical direction so that the principal signal components corresponding to each part are readily identifiable. In this figure, the three semi-transparent overlays highlight the segments of interest: red for samples 0–40 in our sensor (direct air wave), green for samples or time steps within a signal that is transmitted and reaches the soil surface (canopy–surface cluster), and blue for samples and timesteps more than 16.68 ns (subsurface window). Figure 3b shows a single trace (in an A-scan plot) from the same flight, plotted as amplitude (ADC counts) against two-way time, using identical color coding to facilitate cross-reference between the plan view and the trace view.

The first part observed in every trace is the direct air wave (red zone). This peak results from energy propagating across the fixed 0.20 m baseline between the transmitting and receiving antennas and is followed by a weaker ring caused by mutual coupling. Because its timing is governed solely by fixed geometry, this segment provides the reference for time-zero alignment, while its amplitude, governed by the inverse-height relation in Equation (8), serves as an internal gain check (Figure 3b).

Following the direct wave is the canopy–surface cluster (green zone). Early in the season, the cluster appears as a single strong reflection from the soil surface. As the maize canopy develops, multiple short-period reflections emerge from leaves, stems, and tassels; the cluster subsequently weakens as the canopy senesces. The arrival time of the first peak in this zone and the integration of its envelope vary predictably with plant height and canopy water content. Equation (6) indicates that, despite beam widening with altitude, the spatial footprint remains within the 1 m × 1 m validation plots (Figure 3b).

The subsurface window (blue zone) begins at the first local minimum (which is expected to have an arrival time calculated by drone altitude divided by wave velocity in air) below the canopy cluster and extends to the end of the trace. This boundary positions the soil surface at relative time-zero for amplitude-attenuation analysis. When the soil dries, amplitudes in this window decrease slowly; when the soil is wet, higher permittivity delays and attenuates the signal, producing the bowed hyperbolas visible in a. Equation (2) converts two-way travel times to depth, locating the 10, 20, 30, and 40 cm horizons at approximately 145, 170, 195, and 220 samples in our specific study, respectively, while the resolution limit $D_{z}$ given by Equation (8) defines the depth uncertainty. Each segment provides distinct information: peak-to-noise ratio and temporal stability from the direct wave, canopy-water indices from the cluster, and exponential decay plus cumulative energy from the subsurface window. Together, these metrics characterize the complete propagation path depicted in Figure 3a,b and form the basis for the feature-extraction workflow described in Section 3.

## 3. Material and Methodology

### 3.1. Case Studies

**Figure 4:**
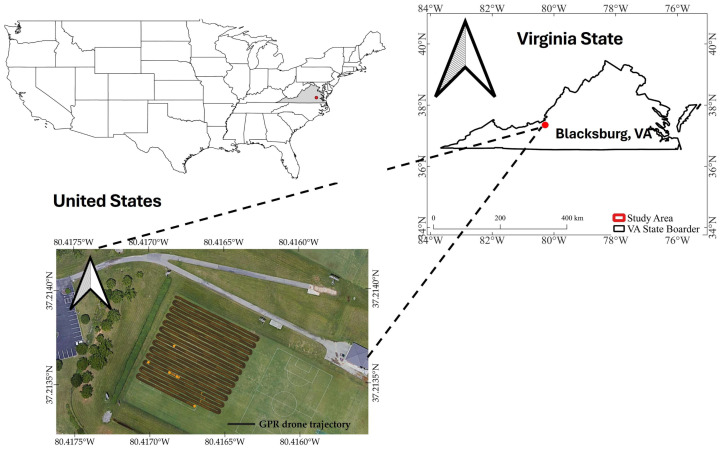
Overview of the location of the first study areaon Virginia Tech campus, with the GPR drone trajectory.

Experiment I (shown in Figure 4) was conducted at the Athletic Field of Virginia Tech’s main campus in Blacksburg, Virginia, USA (37.2284° N, 80.4234° W). This site provided a controlled and uniform environment that was ideal for analyzing drone-mounted GPR signals. The athletic field is a regularly maintained area characterized by an evenly distributed turfgrass canopy. Across the entire site (100 m × 50 m), mowing practices maintain a nearly constant canopy height. The soil texture is classified as clay loam, providing a medium balance of drainage and moisture retention. The field’s surface is nearly flat, with negligible topographic variation, making it particularly suitable for evaluating the effects of flight altitude on radar signal coupling and attenuation. The absence of significant slopes or depressions minimized variability in the antenna-to-ground distance that could otherwise distort the waveform. This site was therefore selected as the baseline location for the first part of the study, which aimed to isolate and quantify the influence of flight altitude and topographic uniformity on the shape, amplitude, and attenuation of signals captured by the sensor.

**Figure 5:**
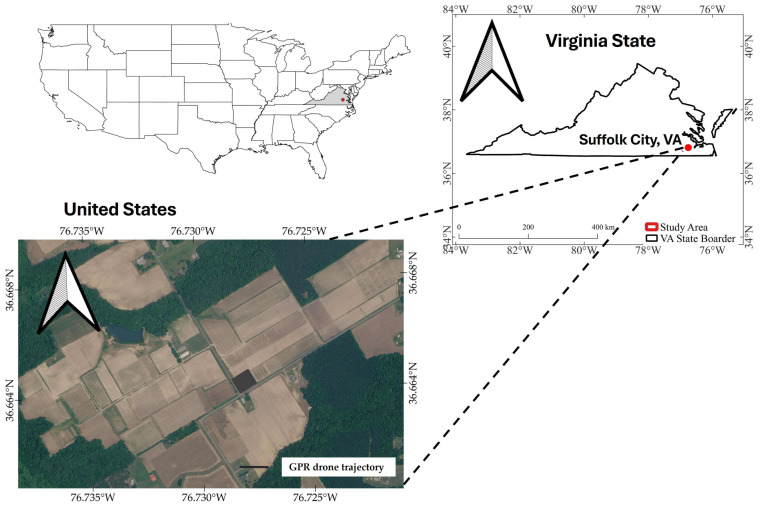
Overview of the location of the second study area, at Tidewater AREC, with the GPR drone trajectory.

The second study area, with a size of 110 m × 48 m (shown in Figure 5), was located at the Tidewater Agricultural Research and Extension Center (TAREC) in Suffolk, Virginia, USA (36.68° N, 76.78° W). This site was planted with maize, active crop growth, allowing for systematic examination of how crop biomass, canopy structure, and water content influence drone-mounted GPR signals. The surface topography is nearly level but with slight undulations typical of field-scale agricultural landscapes. Such mild elevation differences were advantageous for evaluating how small variations in topography and antenna height affect signal arrival time, attenuation rate, and reflection pattern during flight.

### 3.2. Experimental Designs and Drone Flights

#### 3.2.1. Experiment I

Experiment I was conducted at the Virginia Tech Athletic Field, which provided ideal baseline conditions for examining the effects of drone flight altitude on the characteristics of GPR signals. This location was intentionally selected because of its flat topography, homogeneous clay-loam soil, and absence of vegetation, all of which minimize unwanted sources of variability. The experiment was designed to isolate the influence of antenna height above ground level (AGL) on signal strength, attenuation, and subsurface reflection patterns.

To achieve this objective, the GPR system was operated in terrain-following mode, ensuring that the antenna maintained a constant altitude above the ground surface throughout each flight. Data were collected at three distinct altitudes, 1 m, 2 m, and 2.5 m AGL (Table 1), but at the same speed, along identical linear flight paths over the same area of the field. By keeping the flight trajectories consistent, spatial variations in soil conditions or surface roughness were eliminated as potential sources of signal difference. Each flight was conducted within a short time interval (less than one hour total) to ensure that soil moisture and temperature remained stable and to minimize environmental drift between datasets.

Soil moisture in the surface layer (~7.5 cm depth) was measured using a Time-Domain Reflectometry (TDR) probe (Delta-T Devices, Cambridge, UK). Surface hardness and soil compaction were measured using a handheld hardness tester and a cone penetrometer, respectively. A total of 165 ground measurements were collected across the field using a regular grid with approximately 6.5 m spacing. Ordinary kriging was applied to generate spatially continuous maps of soil moisture, surface hardness, and compaction. For the altitude-effect analysis, only GPR traces corresponding to similar soil moisture conditions (the volumetric moisture readings confirmed that all measurement points exhibited nearly uniform water content (variation < ±2%), indicating stable dielectric conditions during all flights.) were selected to minimize the influence of moisture variability on surface reflection amplitude.

#### 3.2.2. Experiment II

Experiment II was conducted at TAREC. This site provided an ideal environment to investigate how minor topographic variations influence signal arrival time and energy attenuation in drone-mounted GPR measurements. Unlike experiment I, which isolated the effects of antenna height in a flat and vegetation-free surface, this study introduced the realistic condition of mild elevation change within an agricultural field while maintaining consistent soil and moisture conditions, even if flight altitude is set to a certain number. Table 1 presented the mission planning parameters in this experiment, with a fixed height of approximately 2.5 m above the takeoff elevation. The flight path extended along a transect with a gradual elevation difference of about 30 cm between the starting and ending points of the mission. Since the reference altitude was measured from the takeoff elevation, small variations in topography resulted in relative changes in antenna height as the drone traversed the field. This setup allowed for the detection of subtle time-delay shifts in reflected radar signals caused by surface elevation changes. The flight was conducted on 15 June, corresponding to the early vegetative stage of maize growth, when canopy biomass and leaf density were minimal. The sparse vegetation ensured that radar wave interaction with plant material was negligible, enabling a clearer interpretation of signal variation due solely to topography and soil surface elevation. The drone maintained a constant forward velocity of 1 m s^−1^, ensuring dense and evenly spaced radar traces along the flight line, suitable for high-resolution B-scan analysis. The altimeter readings collected by the onboard system provided precise, continuous measurements of drone altitude relative to the ground at the moment of each radar transmission. These altimeter-derived height values were later used to correlate local surface elevation with the corresponding signal arrival time in the radar traces, enabling a direct assessment of how even minor vertical differences influence signal delay and attenuation. To ensure that the variations observed in the radargrams were not caused by changes in soil moisture, volumetric water content was measured using Delta-T PR2/6 soil moisture probes preinstalled at the field. The measurements confirmed that soil moisture remained spatially uniform across the flight line, with differences of less than %2 in volumetric water content. This consistency allowed for reliable interpretation of signal variations as the result of elevation changes rather than changes in dielectric properties.

#### 3.2.3. Experiment III

This experiment evaluated how increasing maize canopy biomass during rapid vegetative growth alters the amplitude, waveform shape, and reflect continuity of drone-mounted GPR signals. This study was conducted at the Tidewater AREC (TAREC) and included three UAV–GPR flights collected on 15 June, 30 June, and 11 July, representing low, intermediate, and high canopy conditions, respectively. All flights were performed using the same 500 MHz system at a fixed altitude of 2.5 m above the takeoff reference to maintain consistent acquisition geometry.

This 26-day window was selected to capture the late-vegetative phase, when canopy structure and water content increase rapidly. Based on agronomic growth-stage models

Refs. [25,26], plants progressed approximately from V10 (~1.5 m) in mid-June to VT (~2.2 m) by early July. This period therefore provides a suitable temporal sequence for isolating canopy-driven effects on radar propagation, including increased scattering and attenuation prior to the ground-surface reflection.

Canopy height was quantified using co-registered RGB imagery collected with a DJI Mavic 3T at 20 m altitude (80% front/side overlap). Digital surface models (DSMs) were generated for each date and differenced with a pre-season bare-soil digital terrain model (DTM) to produce plant-height maps. These maps were used to interpret spatial and temporal changes in the GPR response. The main analytical focus of this experiment was the canopy–surface cluster (the time window immediately preceding the ground-surface reflection), which is most sensitive to energy loss and multi-path interactions within the vegetation layer.

#### 3.2.4. Experiment IV

This experiment was also conducted at TAREC, during the later phase of the maize growing season, to examine how changes in canopy and leaf water content, rather than plant height or biomass structure, affect the attenuation and reflection characteristics of airborne GPR signals. All flights were carried out using the same GPR system under identical operational conditions, including a 2 m side distance, constant 2.5 m flight altitude, and a 1 m s^−1^ drone velocity.

Three flights were performed on 20 July, 2 August, and 21 August, corresponding to the Reproductive (R) growth stages of maize (R1 Silking through R3 Milk). By this stage, the plants had reached their maximum structural height, and the canopy architecture remained largely unchanged across the three sampling dates. However, during this period, the vegetation water content (VWC) undergoes substantial physiological variation as the crop transitions from active growth to the grain-filling and early senescence phase [25]. Thus, the dominant factor influencing GPR signal behavior shifted from physical canopy growth to changes in internal water dynamics and dielectric properties of the vegetation. To quantify canopy water content, imagery from a DJI Mavic 3 RGB camera (Shenzhen, China) was collected simultaneously with the GPR flights. Using the red and green spectral bands, the Normalized Difference Greenness Index (NDGI) was calculated for each date following the relationship:

(9) $$NDGI=\frac{G-R}{G+R}$$

where G and R represent the reflectance in the green and red bands, respectively [27]. The NDGI has been widely used as a proxy for leaf and canopy water status and as an indicator of overall crop health and photosynthetic activity [28]. High NDGI values correspond to vigorous, water-rich foliage, while declining NDGI values indicate progressive water loss and physiological stress [29,30]. The NDGI maps were combined with canopy height models derived from previously generated DSM–DTM layers to create a canopy mask, effectively isolating vegetation-covered pixels from soil or inter-row background areas. This allowed the radar response to be analyzed exclusively within the canopy zones where water-content-induced attenuation was expected to dominate.

### 3.3. Methodology

#### 3.3.1. Data Preprocessing and Exploratory Analysis of the Direct Air Wave

Prior to any higher-level signal analysis and processing signals captured in each experiment, a detailed preprocessing and exploratory assessment of the direct air wave segment was conducted to establish a consistent, flight-independent reference for all datasets. Each GPR survey consisted of 512 samples per trace, synchronised with UAV telemetry that recorded altitude, roll, pitch, yaw, and onboard radar board temperature (SF:Temp). These telemetry variables were merged with the radar data using nearest-time alignment within a one-second tolerance. The antenna pair was mounted with a fixed 0.20 m separation in a bistatic configuration, and all flights were executed in terrain-following mode at identical nominal altitudes and waypoints to ensure comparability across datasets.

Raw radar traces were first converted to numerical format, removing any non-numeric or missing values. A pre-trigger baseline was calculated for each trace to remove residual DC offset, followed by a high-pass filter (Butterworth design with order = 4 and a cutoff frequency in Python 3.13 using zero-phase forward–backward filtering to avoid phase distortion) to suppress low-frequency drift. The direct air wave, the initial system pulse transmitted across the fixed antenna separation, was isolated by extracting the first 40 samples of each trace. This segment served as an internal reference pulse whose characteristics depend primarily on aircraft altitude and electronic stability rather than surface or subsurface properties.

Within this window, the global minimum and its two adjacent maxima were automatically identified in every trace to represent the characteristic waveform cycle. For each of these three peaks, the algorithm recorded both amplitude and sample index positions. The spacing between peaks was then computed to assess intra-flight timing stability, and these values were visualized as density distributions to evaluate consistency in pulse shape. Traces that exhibited irregular timing shifts greater than two samples from the signal-to-noise ratios below the fifth percentile were excluded. Outlier traces caused by abrupt changes in roll, pitch, or altitude during sharp turns were also removed based on telemetry-derived thresholds.

To correct minor timing offsets caused by platform motion or clock jitter, a cross-correlation alignment procedure was implemented. For each flight, a median-stacked waveform of the direct air wave was used as a reference template. Every trace was cross-correlated with this template, and temporal alignment was achieved by shifting each trace to maximize correlation. This process effectively reduced timing scatters to within one sample across all datasets. Subsequently, amplitude normalization was performed to remove altitude-dependent gain variation. Since the free-space propagation amplitude decays approximately with the inverse of the antenna height (1/H), each trace was scaled by the ratio of its instantaneous altitude to the flight mean altitude (H/H_0_). This height normalization ensured that signal amplitude differences reflected true electronic or environmental variability rather than geometric spreading effects.

The cleaned and normalized dataset was then used to explore the relationships between telemetry-derived parameters and direct-wave behavior. For each flight, the mean, standard deviation, and variance of altitude, roll, pitch, and radar board temperature were computed to quantify platform stability. Correlation analysis between board temperature and the timing of the direct-wave global minimum revealed a weak but systematic positive slope, approximately one sample for every 2–3 °C increase, suggesting a minor delay due to thermal expansion of antenna components or frequency drift in the radar clock. These trends were visualized using scatterplots and time series plots (as shown in 8–10), confirming that platform motion and thermal variation were the primary sources of residual jitter and amplitude spread.

Finally, cross-correlation alignment and altitude normalization were verified through inter-flight comparison of representative traces. After these corrections, we can confirm that timing drift, altitude variation, and temperature-related gain fluctuations had been effectively mitigated. This established the direct air wave as a stable internal reference, allowing all subsequent analyses of canopy, surface, and subsurface reflections to be interpreted without interference from flight dynamics or instrument drift.

#### 3.3.2. Feature Extraction

In this study, the feature set is built around the physical narrative established in the experimental design section. From the direct-air segment, we record two timesteps, the sample of the first positive half-cycle and the sample of the maximum absolute amplitude, and two amplitude metrics, namely the peak value itself and its signal-to-noise ratio relative to a 40-sample pre-trigger window. Because the geometry of this segment should be constant, these values flag residual timing drift and pulse-to-pulse gain variation; traces that fall outside ±2 samples or ±2 dB are excluded from analysis. However, regardless of the data collection dates, we expect the first part of signals, at least the first positive half-cycle of signals, to show the same feature value, which is meaningless and not significant in our final modeling in any application. From the canopy–surface cluster, we extract the arrival time of the first local maximum, the integrated Hilbert-envelope and 2D continuous wavelet transformation (2D CWT), area over samples 40–120, and the ratio of this area to the direct-wave energy. These quantities are expected to increase with plant height and canopy water content, then decline as the crop matures, providing a proxy for transpiration demand and surface wetness. This is not only expected to show meaningful variation over growing seasons but should also show differences for the dates, showing that there is a high difference in plant water body content in maize plots.

Feature extraction follows a reproducible pipeline implemented in Python. Firstly, we check the location and amplitude of the first positive half-cycle for all the signals captured over the growing season. Then, Hilbert envelope and the four two-dimensional continuous wavelet transformations (CWTs) were computed using Python’s built-in scientific libraries. These features are implemented for each experiment.

#### 3.3.3. Statistical Features

For every trace, we compute first-, second-, and higher-order statistics—mean, median, minimum, maximum, standard deviation, inter-quartile range, skewness, and kurtosis—separately for the direct-air segment and the canopy–surface cluster. Although these quantities are mathematically simple, they summarize the distribution of sample amplitudes in ways that relate directly to plant growth and soil–water status.

In the signal exploratory phase of analysis, the direct-air segment, the waveform shape is governed almost exclusively by the fixed 0.20 m antenna baseline. Consequently, we expect its statistical descriptors to remain essentially constant throughout the season: the mean and variance should fluctuate only within the electronic noise limits of the radar, while skewness and kurtosis should show no systematic drift. Monitoring these values therefore provides a continuous quality check; deviations beyond two standard errors flag traces affected by timing jitter or data-drop artefacts.

The canopy–surface cluster evolves as the crop develops (Experiment I). As biomass increases, additional leaf and stem echoes broaden the distribution: the mean and maximum amplitudes rise, the standard deviation and inter-quartile range increase, and positive skewness emerges as sporadic high-amplitude leaf reflections appear. After tasseling, the cluster envelopes contract as the canopy begins to dry; mean amplitude declines, and the variance returns toward early-season values. We therefore expect a bell-shaped seasonal curve for the mean and variance in the irrigated treatment, with the peak occurring roughly at the mid-July survey, and a similar but lower-amplitude curve in the rain-fed treatment owing to reduced water content in the canopy.

#### 3.3.4. Envelope

To characterize the instantaneous amplitude of each radar segment, we compute the analytic signal via the Hilbert transform. For a real trace $x\left(\tau \right)$, the Hilbert is defined by:

(10) $$ĥ\left(t\right)=\left(\frac{1}{\pi }\right)PV\frac{\int x\left(\tau \right)}{t-\tau }d\tau$$

(11) $$A(t)=sqrt{\left(x(t\right)}^{2}+{ĥ(t)}^{2})$$

The envelope offers two advantages over raw amplitude. First, it is strictly positive, so peak picking and integration are not affected by the sign changes inherent in oscillatory radar signals. Second, it responds linearly to energy variations produced by soil–moisture and canopy–water contrasts, whereas the raw waveform can exhibit constructive or destructive interference that masks true amplitude trends. In the canopy–surface cluster, we integrate the envelope, expecting the integral to rise with increasing biomass and leaf-water content and to decline as the crop senesces. The direct-wave peak validates system stability, the canopy-cluster integral captures temporal changes in aboveground water storage, and the attenuation coefficient, together with depth-resolved energy, detect variations in root-zone moisture.

#### 3.3.5. Peak Info Features

Peaks are other types of locations in signals that contain valuable information. For each identified peak, two quantities are retained. The first is the peak height, which reflects the strength of the underlying reflector. The second is the discrete peak area. In the direct-air segment, only one peak satisfies the condition; its height and area supply a repeatability check that complements the statistical moments described in Section 3.3.3. Within the canopy–surface cluster, multiple peaks appear as the crop matures. The number of peaks, their mean height, and their summed area are expected to rise sharply between V6 and tasseling, then decline as the canopy dries. These trends quantify biomass development and surface-water retention in a manner that the simpler envelope integral cannot capture. The subsurface window often contains several lower-frequency peaks corresponding to interfaces within the root zone.

#### 3.3.6. Wavelet Features

The wavelet-based transformations were applied to GPR B-scans to emphasize structural, textural, and spectral patterns related to canopy interfaces and biomass-induced scattering. Each transform captures complementary aspects of the radar signal—ranging from localized curvature (Laplacian-based operators) to directional texture (Morlet filters) and amplitude modulation (Hilbert envelope). The general principle of these transforms is to convolve the radargram I(x,y) with spatial or temporal kernels that are tuned to specific frequency bands or scales, thereby isolating features of interest while suppressing noise.

- LoG (Laplacian-of-Gaussian)

The Laplacian-of-Gaussian (LoG) operator combines Gaussian smoothing with the Laplacian’s second-order spatial derivative, enhancing edges and curvature while suppressing high-frequency noise. Mathematically, the LoG operator is defined as:

(12) $$LoG(x,y)=\nabla^{2}(G_{\sigma }(x,y)*I(x,y))$$

where $G_{\sigma }\left(x,y\right)=\frac{1}{2\sigma^{2}\pi }e^{-(\frac{x^{2}+y^{2}}{2\pi^{2}})}$ is a Gaussian kernel with standard deviation σ and * denotes convolution. The Gaussian term smooths local fluctuations in the GPR B-scan, while the Laplacian detects regions of rapid intensity change. In UAV–GPR imagery, this transformation is effective for delineating smaller signals, reflected from maize structure. Smaller values of σ emphasize fine-scale detail, whereas larger σ reveal broader canopy to soil surface anomalies.

- DoG (Difference-of-Gaussians)

The Difference-of-Gaussians (DoG) operator approximates the LoG by subtracting two Gaussian-blurred versions of the image with slightly different smoothing scales. It is defined as:

(13) $$DoG(x,y)=G_{\sigma }(x,y)*I(x,y)-G_{k\sigma }(x,y)*I(x,y)$$

where k is typically set to 1.6. This operation functions as a spatial band-pass filter, removing slow background variations and suppressing high-frequency noise while enhancing mesoscale features.

- Gabor/Morlet atom (real, imaginary, magnitude)

The Gabor, or Morlet, transform isolates directional and frequency-specific features by convolving the radargram with a complex sinusoid modulated by a Gaussian envelope:

(14) $$\psi \left(x,y\right)=e^{\left(-\frac{x^{2}+y^{2}}{2\sigma^{2}}\right)}e^{i\left(2\pi f_{0}\right)x+\phi }$$

where $f_{0}$ is the central frequency and ϕ is the phase shift. 

**Figure 6:**
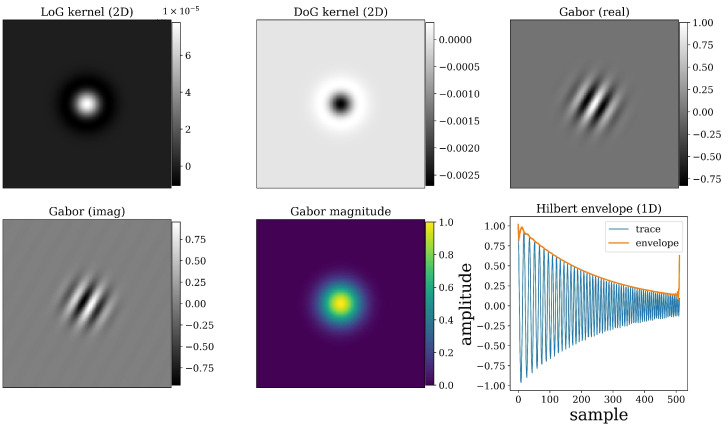
A visualization of envelope feature on 1D signal and Kernels used in 2D CWT.

The convolution of I(x,y) with the complex kernel $\psi \left(x,y\right)$ yields real and imaginary components that describe local oscillatory behavior, their magnitude:

(15) $$M\left(x,y\right)=\sqrt{{\left(R\psi *I\right)}^{2}+{\left(H\psi *I\right)}^{2}}$$

provides a phase-invariant measure of texture energy. In UAV–GPR data, the Gabor transformation is particularly valuable for identifying directional features such as maize row structures. Rotating the kernel orientation enables the extraction of anisotropic patterns that relate to crop geometries. Figure 6 displays the kernels in 2D CWT and 1D envelope feature.

## 4. Results and Discussion

### 4.1. Direct Air Waves (Data Exploratory Analysis)

**Figure 7:**
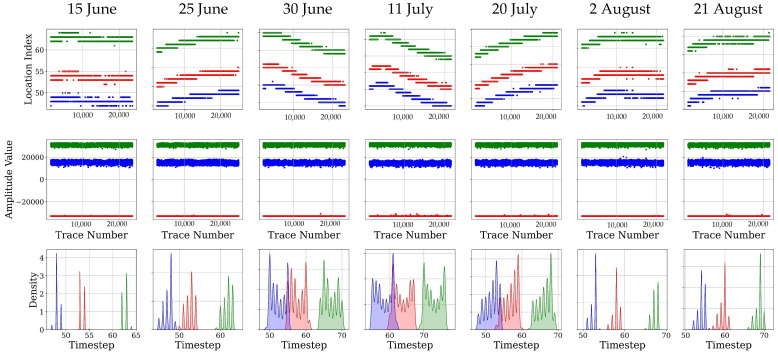
The information (amplitude values and the location in signal) for peaks in the first cycle or part of signals, the upper panels plot the sample indices of the global minimum and its two neighboring maxima, the middle panels plot the corresponding amplitudes, and the lower panels display density estimates of the peak-to-peak spacing.

In this section, the first cycle of GPR signals, captured from our drone, were investigated. The investigation was conducted in three different ways, including finding the location or timestep where the maximum value of amplitude occurred, the maximum amplitude and flight altitude, and canopy and data collection effects on these two parameters to clear any possible variation that might happen, while not expected. Figure 7 shows a subset of drone trajectories in all data collections that we focused on to visualize these three aspects of signal exploration. This part of the field is in the first four irrigated plots. All the signal traces captured in this part of the treatments were explored.

**Figure 8:**
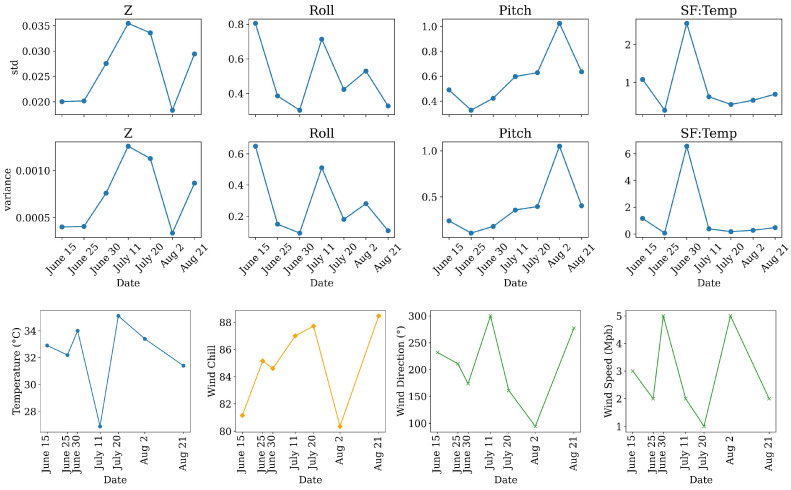
The standard deviation and variance metrics for Flight altitude, Roll, Pitch, and sensor antenna temperature information over the flight, extracted from the GPR sensor trajectory metadata for all dates.

The direct air wave, confined to the first 40samples of every trace, is the system’s internal reference. Because it propagates only across the fixed 0.20 m antenna baseline, its timing and amplitude should depend solely on the aircraft’s altitude and electronic stability. Figure 7 presents the behavior of this pulse on each survey date: the upper panels plot the sample indices of the global minimum and its two neighboring maxima, the middle panels plot the corresponding amplitudes, and the lower panels display density estimates of the peak-to-peak spacing. Figure 8 supplements these data with the standard deviation and variance of altitude, roll, pitch, and board temperature derived from the UAV’s telemetry. Under ideal conditions, the three peaks would appear at identical sample indices on every flight, their absolute positions shifting only with altitude according to the two-way delay of Equation (2), while their amplitudes would follow the inverse-height trend prescribed by Equation (9). The observations conform to these expectations. On 15 June, the first maximum is centered on sample 48; as the nominal flight height increases, the band drifts progressively downward, reaching sample 60 by 21 August. Within any single flight, the peak-to-peak spacing remains constant, confirming that the waveform shape is preserved and that canopy or soil conditions do not influence this segment. 

Although the overall pattern matches theory, the scatter around the mean differs between dates. The widest timing jitter and amplitude spread occur on 11 July and 20 July; altitude and pitch variability, shown in Figure 8, are also highest on these dates, implying that pitch vibration forced larger autopilot corrections and centimetre-scale height excursions. Early in the campaign, on 15 June and 30 June, roll variability peaks and the amplitude envelopes broaden, suggesting that attitude-induced changes in effective antenna separation added a smaller, yet detectable, component of variation. Electronic temperature rises above 25 °C on 11 July, coinciding with the largest amplitude dispersion, but the constant value of the global minimum shows that any thermal drift in the analogue front end remained secondary to altitude effects.

These findings confirm that platform motion and minor clock jitter, rather than other factors such as environmental factors, govern the slight variability of the first cycle. Cross-correlation alignment reduces the timing scatter to within one sample, and normalizing peak amplitude by the measured altitude collapses the inter-flight differences to below one decibel. The 11 July, 30 June, and 20 July datasets, which exhibit the greatest altitude or attitude variance, provide valuable tests for the correction procedure, whereas the 2 August flight, flown under calm conditions with minimal telemetry variance, serves as a benchmark for instrument performance. With the direct wave thus stabilized, subsequent analyses of the canopy–surface cluster can proceed without bias from flight dynamics or temperature-dependent gain.

From Figure 7, three dates of 30 June, 11 July, and 20 July have higher amplitude variation than other dates. It is worth it to the signals captured on these dates that were analyzed in detail. Also, from Figure 8, the GPR sensors had the highest variation of temperature on 30 June, the highest flight altitude variation on 11 July, and the highest pitch variation on 2 August, which is reasonable as the drone flew in unstable wind conditions with a speed of 5 Mph, relatively faster than other dates. Finally, on 20 July, the data collection was conducted in the warmest weather conditions. Thus, all the signal traces, captured on these four dates, are analyzed in detail here. The reason to do this in-detail analysis is to reveal any links or connections between sensor performance, weather condition, and sensor configuration while flying. All missions were programmed at the same nominal height, yet their dynamic behavior differs markedly. In every column the altitude trace drifts downward by 8–10 cm during the first half of the flight before stabilizing. Superimposed on that trend are centimetre-scale oscillations whose magnitude is captured by the standard deviations in the table: σ(Z) rises from 2.2 cm on 30 June to 3.5 cm on 11 July and drops back to 3.1 cm on 20 July. The enlarged height variance on 11 July is consistent with the ±4-sample timing jitter observed in the direct-wave bands (Figure 7) and is the primary driver of amplitude spread on that date.

**Figure 9:**
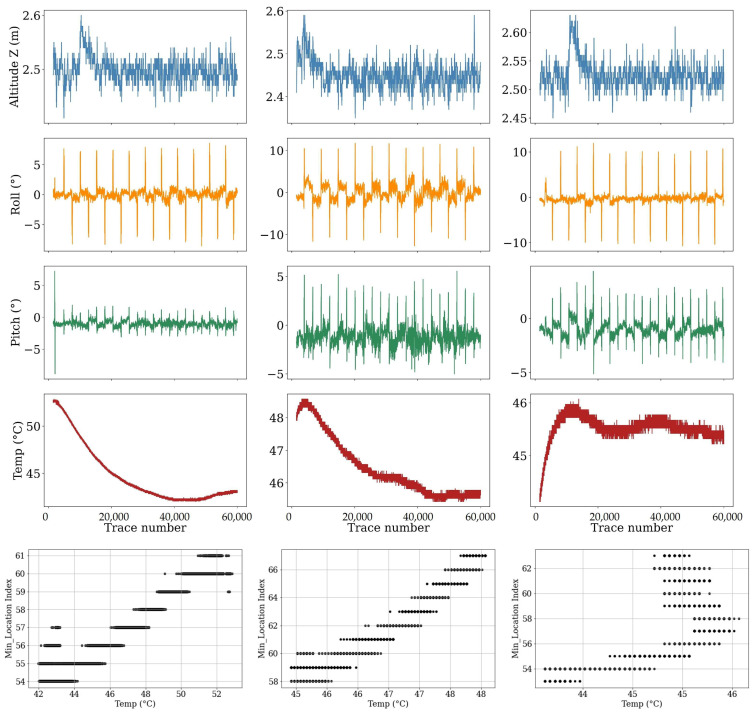
Roll, Pitch, Flight altitude, and temprature of GPR sensor for all the signals collected on 30 June, 11 July, 20 July.

Roll time series (Figure 9) show an expected pattern: the large spikes correspond to the rapid 180° yaw-and-bank maneuvers the drone executes at each end of a pass, while the lower-frequency envelope reflects wind-induced corrections along the swath. Mean roll is close to zero on 30 June and 20 July but climbs to 1.4° on 11 July; the accompanying σ(roll) almost doubles, reaching 0.81°. Pitch behaves similarly, with sporadic downward excursions during deceleration at line ends and upward kicks during acceleration into the following pass. Pitch variability peaks again on 11 July (σ = 0.66°) and is the lowest on 20 July (σ = 0.28°). These attitude excursions alter the effective phase-center spacing of the antenna pair by a few millimeters and add a smaller, yet detectable, component to both timing and amplitude variability.

Sensor-board temperature follows the ambient heat load. From Figure 9, on 30 June, the flight began near solar noon, when the payload had equilibrated at 50 °C and cooled steadily as prop-wash increased convective loss, giving the largest temperature standard deviation (σ = 3.1 °C). On 11 July, the board warmed gradually from 48 °C to 46 °C, while on 20 July, it remained nearly constant (σ = 0.23 °C). Because the global minimum of the direct wave was unchanged across flights, the analogue front end appears insensitive to these temperature swings, indicating that thermal drift is a secondary factor compared with altitude.

Combining the telemetry data with the direct-wave analysis leads to three conclusions. First, the 11 July mission experienced the poorest platform stability and highest σ in altitude, roll, and pitch, and therefore provides the most stringent test of the height-normalization and trace-alignment procedures. Second, the 30 June flight, although flown at a similar mean height, shows larger thermal excursions that can be used to confirm the negligible effect of board temperature on gain. Third, the 20 July survey offers a useful intermediate case: altitude variance is moderate, attitude variance low, and temperature stable, making it an ideal control when evaluating the impact of wind-related motion alone. Detailed examination of these three flights brackets the full range of operating conditions encountered during the season and confirms that the residual variability in the direct air wave is fully accounted for by measurable platform dynamics rather than by canopy or soil effects.

The scatterplots in the last row of Figure 9 add a final result from the direct-wave analysis. Although altitude variance was identified as the principal driver of timing scatter, the monotonic trend between SF:Temp and minimum-sample index shows that thermal expansion of the antenna housing or small shifts in the radar-clock frequency contribute an additional, temperature-dependent delay. The slope is modest, with about one sample for every 2 °C on 30 June and less than one sample per 3 °C on the other dates, but it is consistent across all three flights. This systematic behavior confirms the need to reference every trace to the same internal template rather than relying on a fixed-sample window.

**Figure 10:**
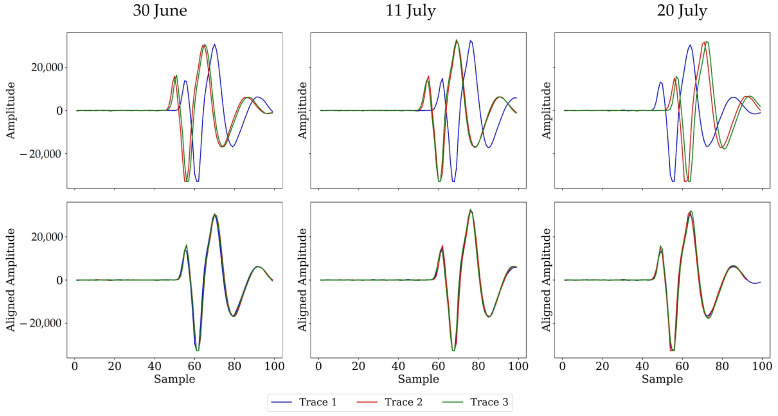
Effectiveness of trace alignment. Top row: raw first-cycle waveforms from three randomly selected traces on 30 June, 11 July, and 20 July. Bottom row: the same traces after cross-correlation alignment and height normalization. Alignment collapses the time drift to within one sample and removes the altitude-related gain spread, bringing all three traces into near-perfect register.

Demonstrates that the adopted correction procedure resolves both sources of variability. Before alignment, the three example traces on each date exhibit the amplitude divergence and timing offsets diagnosed in Figure 8, Figure 9 and Figure 10; after alignment, the peaks coincide, and the amplitude envelopes overlay almost exactly. The remaining sub-sample differences lie well below the intrinsic ADC jitter measured in laboratory tests, indicating that platform motion, altitude drift, and temperature effects have been effectively removed. With the direct air wave now serving as a stable, temperature- and height-normalized reference, the subsequent analyses of the canopy–surface cluster (Section 4.2) and the subsurface window (Section 4.3) can be interpreted without concern that instrument or flight artefacts contaminate the agronomic signal of interest.

### 4.2. Results of Experiment I (AGL Effect)

**Figure 11:**
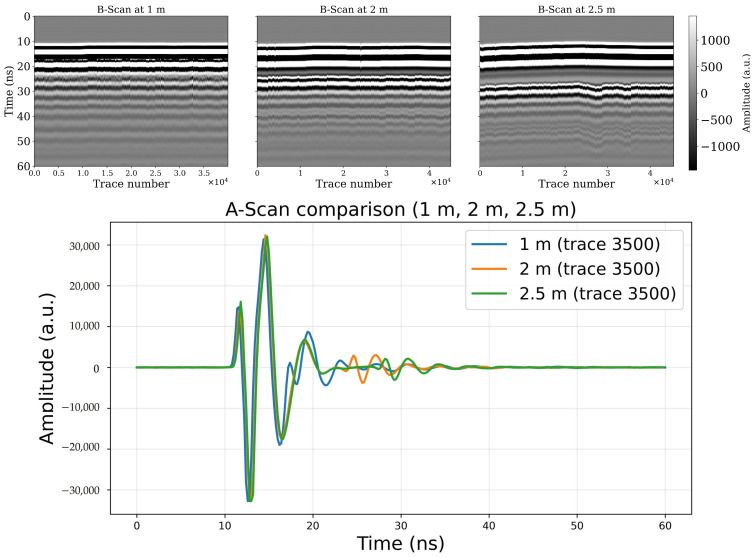
Effect of flight altitude on drone-mounted GPR signals over a flat, biomass-free soccer field. (**Top row**) Representative B-scans at each altitude illustrate the shift in soil surface arrival time. (**Bottom row**) A-scan comparison from trace 3500 highlights the direct wave, soil reflection, and attenuation differences across altitudes, showing both delayed arrival and reduced amplitude with greater flight height.

From the theory, the travel time to the soil surface scales with the path length through air, since the pulse propagates at speed c in free space (Equation (2)). Raising the antennas delays the soil reflection by Δt proportional to the added height. At the same time, free-space spreading reduces signal strength according to the inverse-square law (Equation (1)), and the received amplitude scales approximately as 1/H (Equation (8)). Thus, with increasing AGL, we expect: (i) a systematic shift in arrival time of the surface reflection, (ii) reduced energy in both the direct and reflected components, and (iii) a higher apparent attenuation rate when traces are normalized by altitude. The soccer-field flights allow us to confirm these predictions in practice, showing clearly how altitude affects the baseline reference wave, the soil surface return, and the subsurface window even in the absence of canopy or soil heterogeneity. In Figure 11, the B-scans (top row) show the soil-surface reflector shifting progressively to later time as altitude increases, while the deeper wave train weakens. This agrees with the free-space leg of the path: increasing the air gap by H adds a two-way delay $\Delta t\;\approx \;2H/c\;(with\;c\approx 0.3\;{m\;ns}^{-1}),$ and reduces amplitude by geometric spreading (amplitude ∝ 1/H, power $\propto 1/H^{2}$). The A-scan overlay (bottom row) makes both trends explicit: relative to 1 m, traces collected at 2–2.5 m show a later soil-surface arrival and a lower peak amplitude, after which the post-surface wavetrain decays more quickly into the noise floor. Because the site was level, biomass-free, and observed under uniform moisture, the contrasts are attributable to altitude rather than environmental differences.

**Figure 12:**
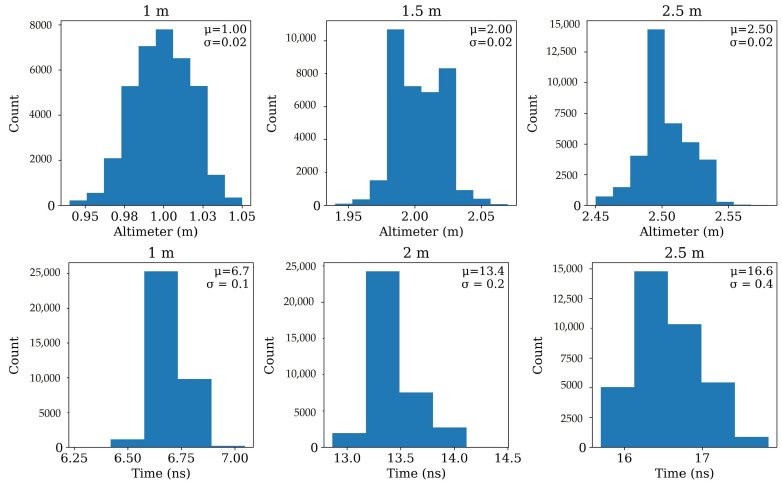
The first-row subplots: altimeter reading at the time of received signal, the second row subplots: two-way travel time of reflection from soil surface calculated for all the traces in each flight.

However, the results in Figure 11 came from an average of a few signals from the same area in the soccer field. To have in-detail observation on all the traces captured in the flight missions, we checked the distributions of two-way travel time and AGL vibration of the drone while flying over the area. Figure 12 shows perfectly the distribution of these two variables for the three flights. The top row summarizes altimeter readings for every trace in each flight. All three histograms are narrow and centered at programmed heights (means ≈ 1, 2, and 2.50 m with σ≈0.02 m), confirming that terrain following and platform control kept altitude jitter small. The bottom row shows the corresponding distributions of the two-way travel time of the soil-surface reflection picked on each trace. The modes align with the free-space prediction: ≈6.7 ns at 1 m, ≈13.3 ns at 2 m, and ≈16.6–16.7 ns at 2.5 m (close to 2H/c). The standard deviation grows slightly with altitude (≈0.1 ns at 1 m, ≈0.2 ns at 2 m, ≈0.4 ns at 2.5 m), which is consistent with the fact that a longer air path makes the pick mildly more sensitive to small height fluctuations and time-zero jitter. The tight alignment of these histograms demonstrates that the surface-arrival picker is consistent across flights and that the experiment successfully isolated the altitude effect.

**Figure 13:**
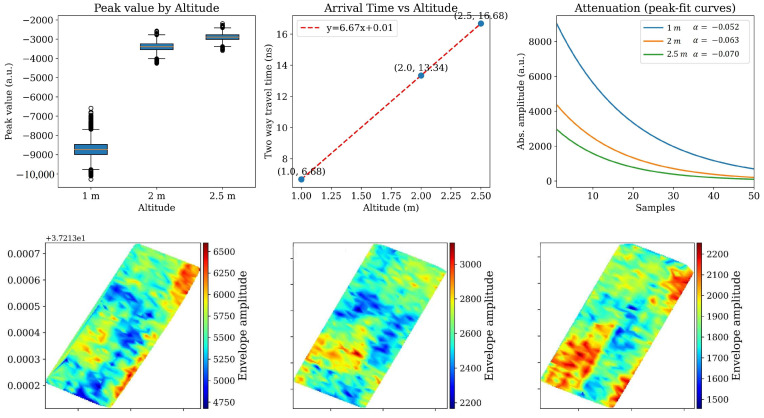
Summary of the effects of drone altitude on GPR signal characteristics. (**Left**) Boxplots of soil-surface reflection amplitudes at 1 m, 2 m, and 2.5 m altitudes, (**Middle**) Relationship between drone altitude and two-way travel time of the ground reflection, (**Right**) Exponential attenuation curves fitted to peak values.

Figure 13 provides a focused evaluation of how increasing flight altitude systematically influences GPR signal properties. To ensure that the observed changes are attributable to altitude alone, we restricted the analysis to locations within the soccer field that shared uniform soil conditions. Moisture content was measured with a TDR probe, and only traces collected over areas with comparable subsurface moisture were used for the attenuation-rate calculations. By controlling soil variability, the figure isolates three complementary aspects of the altitude effect: (i) the reduction of reflection strength with height, (ii) the linear increase of soil-surface arrival time (the red dash line equation y = 6.67x + 0.01), and (iii) the steepening of apparent post-surface decay. Together, these results highlight how changes in antenna height alone can influence both the amplitude and timing of GPR signals under consistent field conditions.

The left panel of the first row compares the soil–surface peak amplitude across altitudes. Median magnitudes are largest at 1 m and substantially smaller at 2 m and 2.5 m, with a much narrower spread at the higher altitudes. This monotonic decrease matches geometric spreading: with everything else held constant, lifting the antenna reduces the amplitude roughly as 1/H. Outliers at 1 m are mostly tied to endpoints and occasional attitude corrections, which have proportionally larger influence when the antenna is close to the ground. The middle panel plots arrival time versus altitude using the flight means. The points fall on a line with slope ≈6.67 ns m^−1^, which is the theoretical value of 2/c. The near-zero intercept indicates negligible fixed delay in the picking relative to time-zero. This linearity confirms that the measured delays are dominated by the free-space segment and validates the timing model used elsewhere in the analysis. The right panel shows exponential attenuation curves fitted to peak sequences in a fixed post-surface window (50 samples) after averaging ±10 neighboring traces around a representative trace number. The fitted log-linear slopes (per-sample) become more negative with altitude (α ≈ −0.052 at 1 m, −0.063 at 2 m, −0.070 at 2.5 m). Because the soil did not change between flights, we interpreted these as apparent attenuation: lifting the antenna reduces the initial amplitude that seeds the subsurface wave train and increases the relative contribution of system noise and residual ringing within the fixed window, yielding a steeper effective decay. This behavior is exactly what we would expect when geometric spreading in the air path combines with a constant soil loss term over a finite window. The subplots in the second row of Figure 13 demonstrate that, although the envelope amplitude and attenuation rate vary slightly with changes in flight altitude (AGL), the spatial pattern and relative differences in envelope values across the field remain consistent. This stability suggests that the observed variations are more likely related to inherent soil properties, such as moisture content, hardness, or compaction—rather than altitude-induced effects.

### 4.3. The Results of Experiment II (Topography Effect)

Experiment II was designed to assess how slight topographic variations across the Tidewater field affect the timing and energy of the ground surface reflection in air-launched GPR data. Although the drone was programmed to maintain a nominal altitude of 2.5 m above the takeoff elevation, the terrain exhibited a gradual slope of less than 30 cm, resulting in measurable changes in the actual antenna height above ground level (AGL) during flight.

**Figure 14:**
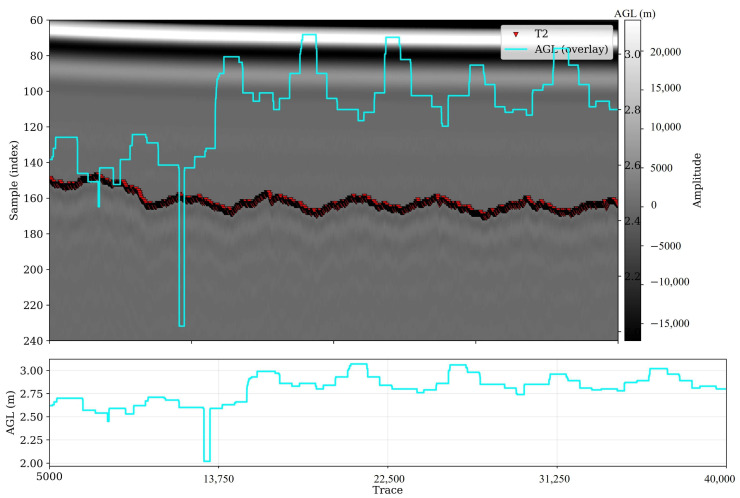
Subset of GPR signals captured on 15 June; T2 is the arrival time to soil surface and blue line graph: altimeter reading.

Figure 14 presents a subset of the B-scan captured on 15 June, in which the arrival time of the ground surface reflection (denoted T2) is superimposed on the raw GPR amplitudes along with the altimeter-derived AGL readings (cyan). The upper panel clearly illustrates that as the terrain elevation increases, the arrival time (sample index) of the soil surface reflection shifts upward (i.e., occurs earlier), while in lower portions of the field, where the drone effectively flies higher, the reflection arrives later in time. The consistent inverse relationship between AGL and T2 across the swath confirms that the two-way travel time of the radar pulse closely follows the geometric height variation of the platform. The bottom subplot of Figure 14 further quantifies this pattern, showing smooth fluctuations in AGL between 2.0 m and 3.0 m, reflecting the minor slope across the field. This agreement validates the accuracy of the onboard altimeter as a proxy for instantaneous antenna height.

**Figure 15:**
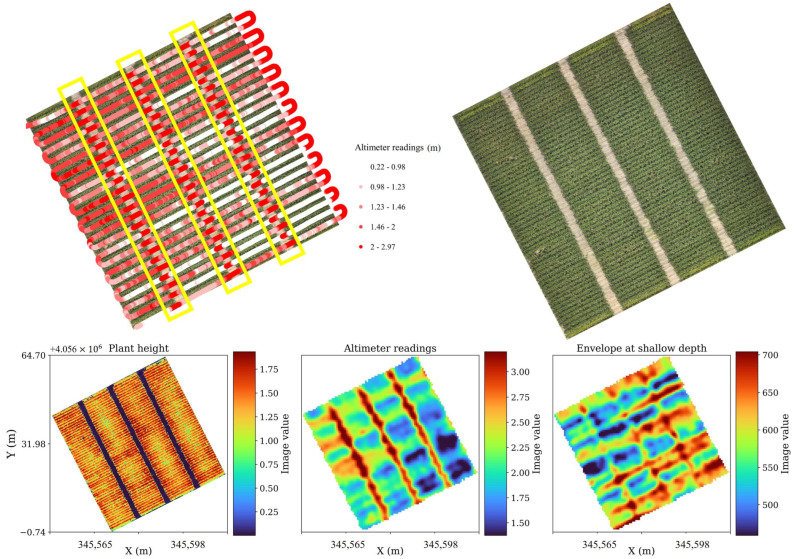
Plant height, altimeter readings, and envelope of amplitude from shallow depth on GPR signals of 15 June, the yellow rectangles show highest value of altimeter readings where there is bare soil.

Spatial visualization of the altimeter data and GPR-derived attributes further supports these relationships. Figure 15 shows the flight paths over the field (upper panels) and the derived maps of plant height, altimeter readings, and shallow-depth envelope amplitude (bottom row). The three maps exhibit strikingly similar spatial patterns: regions of higher altimeter readings correspond to areas of slightly lower envelope amplitude, while lower AGL zones show stronger reflected energy. This inverse correlation confirms that the signal amplitude decreases with increasing antenna height, consistent with geometric spreading and partial attenuation of the emitted wavefront. In addition, the coherent banding visible in both the AGL and envelope maps reflects the systematic pattern of flight lines, demonstrating that the altitude variations are primarily due to surface elevation rather than sensor noise or control instability.

**Figure 16:**
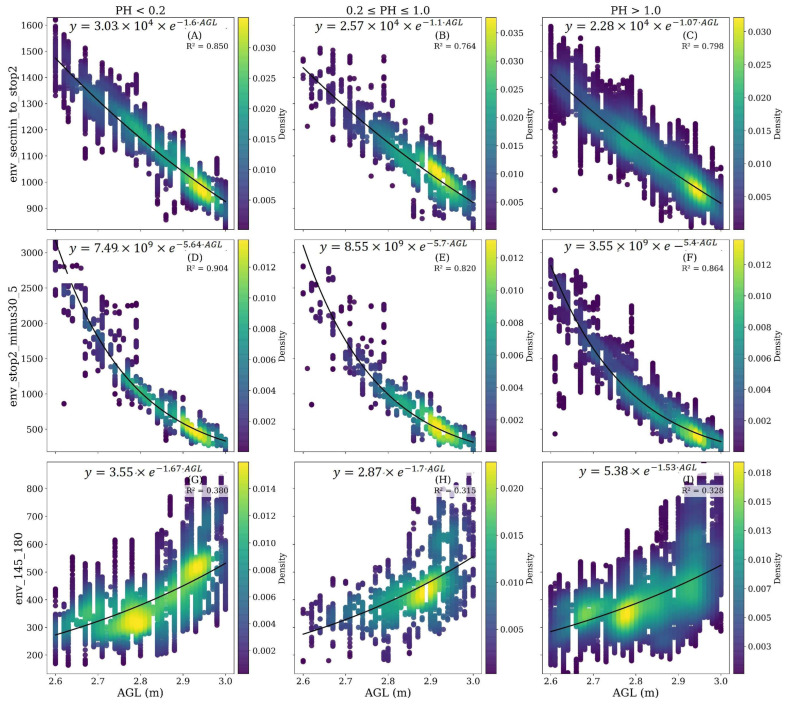
The scatterplot of calculated envelope Vs AGL for three time ranges of, classified by plant height.

To further quantify the influence of AGL and canopy structure on signal behavior, envelope amplitudes from three representative time windows were analyzed against altimeter-derived height across three plant height categories (low, medium, and high). The scatterplots in Figure 16 summarize these relationships. In all panels, the envelope amplitude exhibits an exponential decay trend with increasing AGL, as modeled by the fitted function $E=Ae^{-kAGL}$, where A is the reference amplitude and k is the attenuation coefficient. The fits show strong coefficients of determination (R^2^ > 0.80) for shallow and mid-depth reflections (Figure 16A–F), indicating that altitude-dependent energy loss dominates signal variation in these layers. At deeper time windows (Figure 16G–I), the correlation weakens (R^2^ ≈ 0.3–0.4), suggesting that noise and subsurface heterogeneity play a greater role at later arrival times. A comparison across plant height classes reveals that attenuation is steeper at higher canopy heights (right column of Figure 16), implying that biomass presence slightly amplifies energy loss even within the same geometric height range. Nonetheless, the persistence of the exponential decay pattern across all plant height categories confirms that altitude and topography remain the primary drivers of signal amplitude and arrival-time variation in this dataset. This relationship motivated us to design experiment III, where we analyze biomass and canopy structure changes over time series.

### 4.4. The Results of Experiment III (Crop Structure Effect)

Before evaluating the GPR signal behavior under varying biomass and moisture conditions, the temporal dynamics of the maize canopy were analyzed using plant height (PH) and Normalized Difference Green Index (NDGI) derived from drone-based RGB imagery. These parameters provide complementary insights into canopy structural development and its physiological state during the growing season.

**Figure 17:**
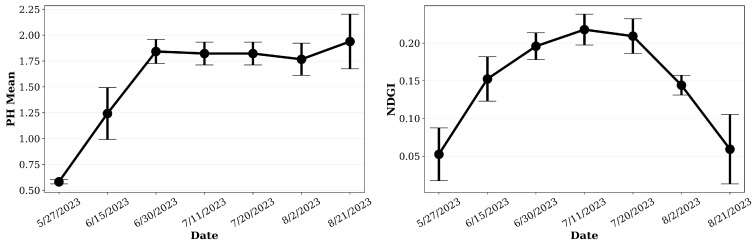
The plant height and canopy NDGI variation over the crop growing seasons.

Figure 17 summarizes the evolution of mean PH and NDGI values from late May to late August. The PH curve shows a rapid increase between 1 June and 11 July, corresponding to the vegetative growth phase when stem elongation and leaf expansion occur at their maximum rate. By mid-July, plant height plateaued at approximately 2.1 m, marking the transition from vegetative to reproductive stages. This plateau indicates that structural growth had largely stabilized, with subsequent variations mainly reflecting minor lodging or measurement uncertainty. In contrast, the NDGI trajectory exhibits a distinct rise and fall pattern: the index increases steadily from early June to a peak in mid-July, followed by a gradual decline through August. This pattern reflects the accumulation and subsequent reduction of vegetation water content (VWC) and leaf chlorophyll, consistent with the physiological progression from vigorous photosynthetic activity to senescence and water redistribution toward reproductive organs. The temporal alignment between the NDGI peak and the PH plateau emphasizes that July 11 represents the period of maximum canopy density and water content, when GPR signal attenuation effects are expected to be strongest.

**Figure 18:**
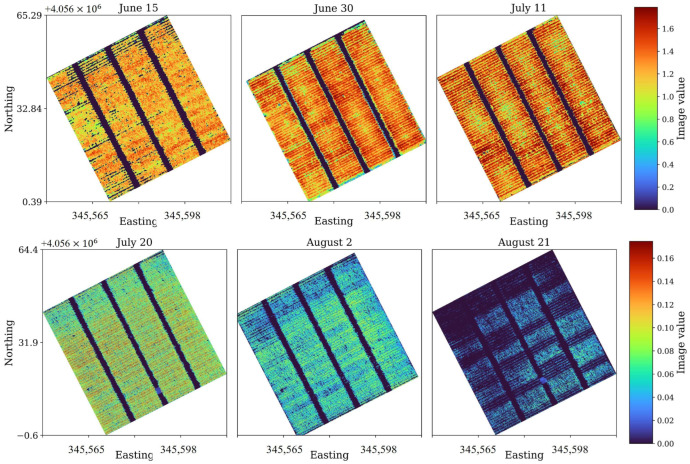
Plant height and NDGI spectral index of plot in three dates for experiment II and experiment III.

Spatial distributions of PH and NDGI across the study plot further illustrate these trends (Figure 18). The upper three panels (15 June, 30 June, and 11 July) show progressively denser and more uniform canopy structure, with plant height increasing from approximately 1.2 m to 2.2 m. Concurrently, NDGI values rise from 0.12 to 0.21, highlighting enhanced canopy greenness and water absorption capacity. These spatial patterns reveal subtle within-field variability along the flight lines, likely influenced by micro-topography and soil moisture distribution. The lower three panels (20 July, 2 August, and 21 August) capture the post-peak phase of canopy development. Here, NDGI values drop sharply—from ~0.18 to below 0.05—while plant height remains nearly constant. This divergence indicates that while the physical canopy structure remained intact, the leaf water content and photosynthetic activity declined rapidly due to the onset of senescence. The pronounced spectral shift from bright orange to dark blue in the NDGI maps visually confirms this physiological transition.

The selection of the three GPR flight datasets between 15 June and 11 July is specifically designed to isolate and characterize the maximum influence of rapid vegetative biomass accumulation on the air-launched GPR signal. This 26-day period captures the corn plant’s transition through the late vegetative stages, likely from approximately V10 (1.5 m height) through V(n) to Tasseling (VT) (2.2 m height), which marks the phase of maximum stem elongation and leaf area development [25]. During this window, the stalk increases in diameter and height at its fastest rate, and the Vegetation Water Content (VWC) within the leaves and stalks approaches its seasonal peak (typically ≈ 85% fresh weight). As the dielectric permittivity (ε) of the vegetation is dominated by water, this critical period represents the most aggressive, non-linear increase in the GPR signal attenuation coefficient. The selected flights, therefore, offer a high-resolution temporal series for quantifying the direct link between the rate of canopy structural growth and the corresponding deterioration of the Ground Surface Reflection (GSR).

**Figure 19:**
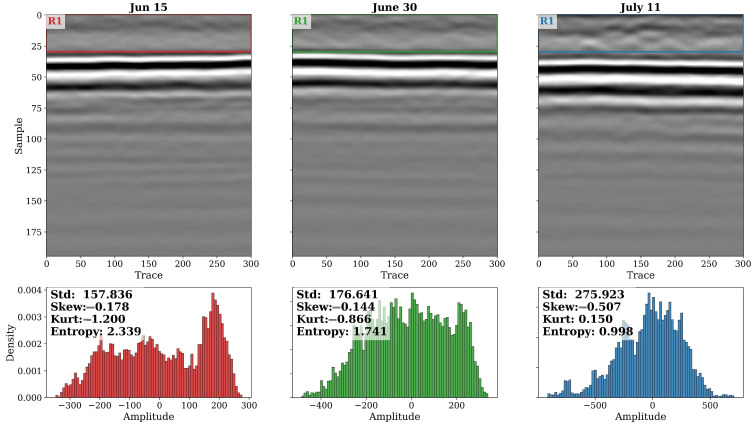
B-scan plots of GPR signals, captured on the dates 15 June, 30 June, and 11 July.

Figure 19 presents B-scans and amplitude histograms of GPR subsets captured on these three dates. The 15 June profile exhibits a well-defined, high-contrast reflection from the soil surface with minimal signal scattering above it. The amplitude distribution shows a moderate spread (Std = 157.8) and relatively low entropy (2.34), indicating a stable, low-noise waveform typical of bare or early-stage canopy conditions. By June 30, the reflection band becomes slightly broader and less sharply defined, accompanied by a wider amplitude range (Std = 176.6) and reduced entropy (1.74). This change reflects the onset of canopy interference and the increased dielectric heterogeneity caused by expanding leaves and stems. By 11 July, when plants reached their maximum vegetative height, the GPR signal shows pronounced amplitude reduction and vertical smearing, characteristic of strong attenuation and multiple scattering within the canopy layer. The histogram broadens further (Std = 275.9), and the distribution becomes more symmetrical (Skew ≈ −0.51, Kurt = 0.15), indicating the loss of distinct high-energy peaks associated with the ground return. The entropy drop to ≈1.00 confirms a marked reduction in overall signal complexity due to energy damping and coherence loss. Together, these trends demonstrate a progressive weakening of the ground reflection and increased signal diffusion consistent with rapid biomass accumulation and rising vegetation water content.

**Figure 20:**
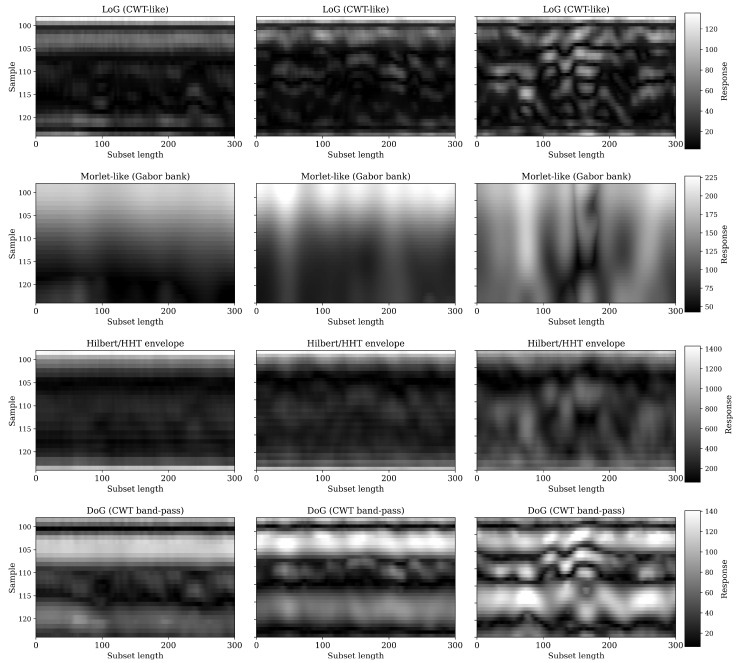
The results of four CWT transformations on the same size of subsets, locations from three dates of 15 June, 30 June, and 11 July.

To further investigate the structural and textural evolution of the signal, four complementary 2-D wavelet transformations were applied to equivalent subsets from each date: Laplacian-of-Gaussian (LoG), Difference-of-Gaussians (DoG), Morlet (Gabor-like) bank, and Hilbert (HHT) envelope (Figure 20). The LoG responses show a clear temporal progression—from weak curvature contrast on 15 June to highly textured, spatially complex patterns by 11 July, revealing the emergence of fine-scale amplitude irregularities as canopy scattering intensifies. The DoG band-pass results exhibit a similar enhancement of mesoscale structure, confirming that the mid-frequency energy content of the GPR signal increases with vegetation development.

The Morlet-like (Gabor) transform provides a spectral view of this process: its amplitude increases steadily from 15 June to 11 July, particularly in mid-depth regions, indicating stronger localized oscillations and frequency-dependent scattering. The Hilbert envelope follows a comparable pattern, showing a gradual decline in mean amplitude and increasing variability, which together reflect both attenuation and multi-path interference within the canopy. Collectively, the four transformations reveal that vegetative growth introduces progressively higher spatial and spectral heterogeneity into the radargram, reducing the coherence of the ground reflection and redistributing signal energy across multiple time–frequency scales.

**Figure 21:**
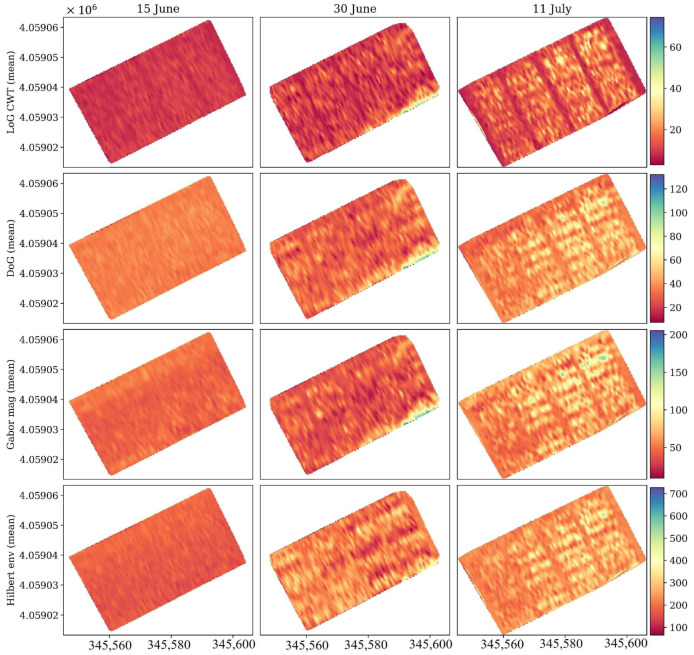
Maps of features obtained from the four transformations.

Spatial maps of these derived features, computed for the entire field (Figure 21), provide a two-dimensional perspective of this attenuation process. For all four transforms, the maps evolve from relatively uniform distributions on 15 June to visibly banded and heterogeneous textures by 11 July. The LoG and DoG maps highlight spatially consistent zones of increased curvature and mid-scale energy, corresponding to canopy row patterns and biomass gradients. The Gabor magnitude and Hilbert envelope maps both show a systematic decline in mean intensity and a growth in localized high-frequency components, indicating that as the canopy thickened, surface reflections weakened while near-surface scattering increased.

### 4.5. Canopy Water Content (CWC) Effect (Experiment IV)

**Figure 22:**
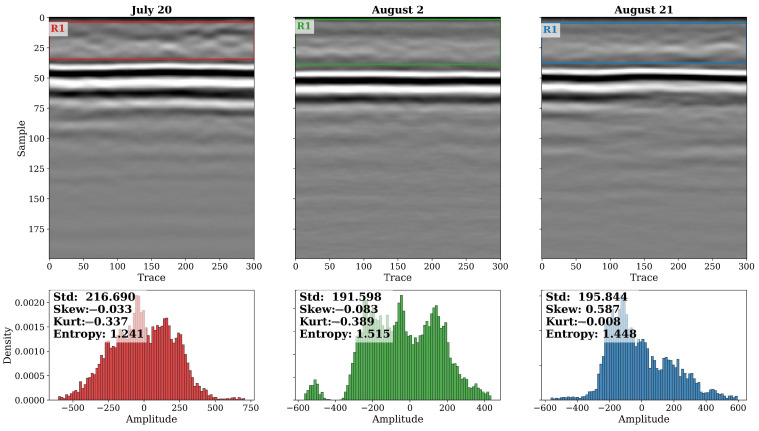
B-scan plots of GPR signals, captured on the dates 20 July, 2 August, and 21 August.

The final experiment focused on the late reproductive phase of the corn crop, when plant height had already stabilized and the major source of variation in canopy dielectric properties was due to changes in water content. As shown in Figure 22, B-scan profiles captured on 20 July, 2 August, and 21 August reveal distinct shifts in signal texture and amplitude distribution despite identical flight altitudes and soil conditions.

The mean amplitude remained comparable among the three dates, but the statistical descriptors, particularly standard deviation (Std) and entropy, demonstrated systematic trends associated with canopy moisture decline. On 20 July, when canopy water content was at its seasonal peak, signal variability was the highest (Std = 216.7), but entropy remained relatively low (1.24), indicating uniform attenuation across the field. By 2 August, entropy increased to 1.51, suggesting a more heterogeneous pattern likely caused by patchy drying and uneven moisture redistribution. On 21 August, both skewness and kurtosis values changed sign, with entropy remaining high (1.45), reflecting the emergence of localized weak reflections as the canopy dried and dielectric contrast increased.

**Figure 23:**
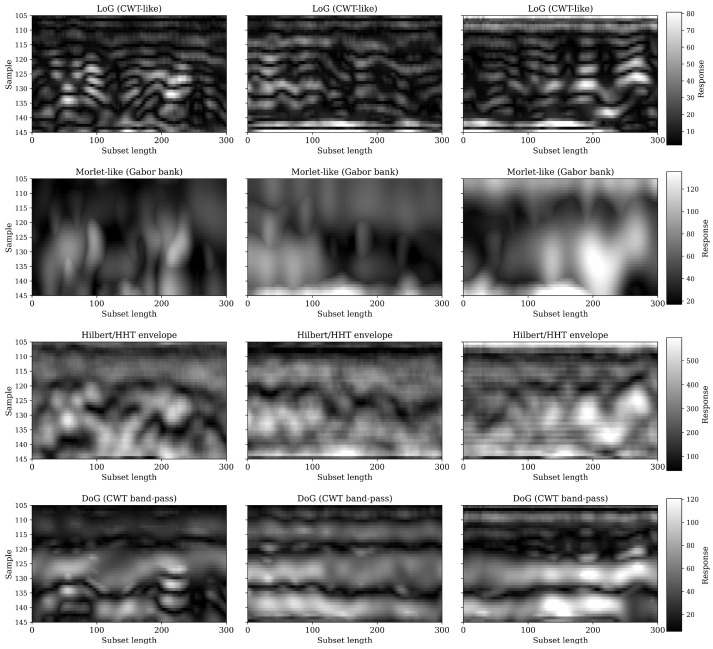
The results of four CWT transformations on the same size of subsets, locations from three dates, 20 July, 2 August, and 21 August.

The 2D transformations in Figure 23 provide complementary evidence of these seasonal dynamics. The LoG and DoG filters emphasized the gradual loss of continuity in strong horizontal reflections from 20 July to 21 August, illustrating a reduction in the coherence of the ground surface reflection. The Morlet (Gabor) transform produced progressively lower response magnitudes, indicating decreasing signal energy at the dominant frequency bands as vegetation water content diminished. Meanwhile, the Hilbert envelope showed weakening in the amplitude envelope over time, capturing the overall reduction in backscattered signal energy caused by declining canopy permittivity. These transformations collectively highlight the sensitivity of multi-scale wavelet responses to water-induced dielectric variations within the canopy and topsoil interface.

**Figure 24:**
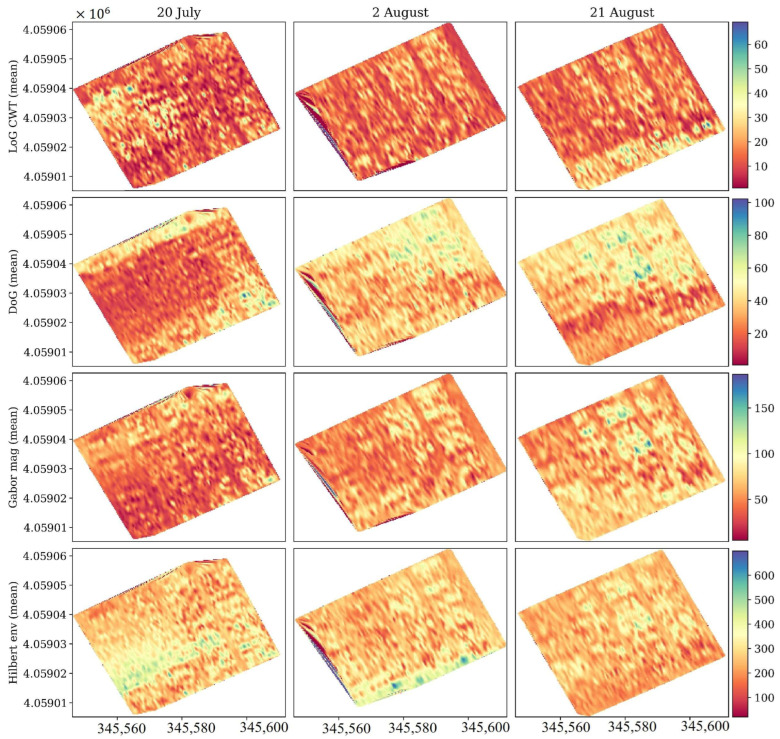
Maps of features obtained from the four transformations on three dates, 20 July, 2 August, and 21 August.

Spatially, the mean feature maps in Figure 24 reveal coherent field-scale patterns that closely correspond to canopy water content derived from NDGI (see Figure 18). On 20 July, all four transformations (LoG, DoG, Gabor magnitude, and Hilbert envelope) displayed strong, spatially uniform intensities consistent with a dense and water-saturated canopy. By 2 August, the patterns became more heterogeneous, with alternating high- and low-intensity zones aligned with row orientation, suggesting variable drying rates between inter-row and intra-row regions. By 21 August, the overall magnitude of all transformations decreased sharply, and the contrast between rows weakened, an indication that the canopy had transitioned toward physiological maturity, with reduced water content and dielectric homogeneity.

## 5. Conclusions

This study presented a systematic analysis of drone-mounted Ground Penetrating Radar (GPR) signals collected with a 500 MHz antenna to understand how flight altitude, topography, vegetation structure, and canopy water content influence the amplitude, timing, and overall quality of radar reflections. Through four targeted experiments conducted over controlled turf and agricultural fields, we were able to isolate the individual and combined effects of these factors and identify the key sources of uncertainty that arise when operating an airborne GPR system over vegetated surfaces. Experiment I, conducted over a bare, flat athletic field, demonstrated that variations in altitude alone can strongly affect signal amplitude and attenuation rate, following an exponential decay trend consistent with theoretical spreading loss. These results provided an important baseline for understanding signal energy loss under ideal, vegetation-free conditions.

Experiment II, performed over the Tidewater corn field with minor topographic variation, showed that even subtle elevation differences of less than 30 cm can cause measurable two-way travel time shifts and amplitude distortions. When the GPR antenna’s true height above ground was corrected using onboard altimeter data, signal arrival times aligned accurately with terrain elevation, confirming that real-time altitude correction is essential for reliable depth estimation and amplitude normalization. Experiment III focused on the vegetative growth period between mid-June and mid-July, capturing the phase of maximum biomass accumulation. During this period, plant height increased from approximately 1.5 m to 2.2 m, and vegetation water content approached its seasonal maximum. The results clearly showed that as canopy density and water content increased, GPR signal amplitude and coherence declined sharply, while wavelet and envelope transformations revealed more complex spatial and spectral textures. These changes confirm that vegetation not only attenuates radar energy but also introduces multi-path scattering that reshapes the signal’s internal structure.

The final experiment, covering late-season flights from 20 July to 21 August, revealed a different form of signal degradation. Although plant height remained stable, NDGI and canopy water content declined sharply, causing variability in the dielectric environment. During this stage, signal attenuation became less dominated by structural obstruction and more by dynamic changes in canopy moisture, which affected the dielectric contrast between vegetation and air. Together, these findings highlight that both the quantity and the water content of biomass strongly influence GPR signal propagation in agricultural environments.

Despite these advances, several limitations remain. First, the experiments were limited to a single radar frequency (500 MHz) and a narrow range of altitudes; extending measurements to higher and lower frequencies would better quantify depth-dependent attenuation. Second, while altimeter readings were effectively corrected for geometric variation, soil moisture and temperature gradients were measured only at discrete points and could not fully capture sub-field heterogeneity. Third, biomass characterization relied primarily on NDGI and plant height; integrating thermal infrared and hyperspectral indices could strengthen the link between vegetation water status and radar signal loss. Finally, signal analysis was limited to two-dimensional and one-dimensional transforms; future work could explore three-dimensional CWTs or deep learning approaches (e.g., 1D-CNNs or attention-based models) to automatically extract spatio-temporal features from full B-scan datasets. Future research should aim to couple airborne GPR and optical/thermal sensors within a unified modeling framework, enabling more accurate estimation of soil and canopy dielectric properties under changing environmental conditions. Controlled field trials that manipulate irrigation or biomass water content could further validate the observed relationships between canopy structure and radar attenuation. In addition, expanding experiments across different crop types, soil textures, and seasonal stages will be essential for developing generalized correction models applicable to real-world agricultural monitoring. In summary, this work provides a first step toward understanding how environmental and operational factors interact to shape air-launched GPR signal behavior. By quantifying the separate effects of altitude, terrain, biomass, and canopy water content, it lays the groundwork for more accurate soil moisture estimation and subsurface mapping from UAV platforms. With further refinement, drone-based GPR could evolve into a powerful, non-invasive tool for precision agriculture, offering new insights into soil–plant–atmosphere interactions beneath living canopies.

## Figures and Tables

**Table 1:** The drone flights conducted in the experiments.

Experiment	Number of Flights	Flight Altitude (m)	Side Distance (m)	Drone Speed
I (AGL effect)	3	1, 2, 2.5	2	~1 m/s
II (Topography effect)	1	2.5	2	~1 m/s
III (Biomass effect)	3	2.5	2	~1 m/s
IV (Water content effect)	3	2.5	2	~1 m/s

## Data Availability

The data presented in this study are available on request from the corresponding author.
